# Comparative Study of Vibration Response in Steel and Braided-Carbon-Fiber Bicycle Handlebars: A Numerical-Experimental Approach with Various Sensors

**DOI:** 10.3390/s24061767

**Published:** 2024-03-08

**Authors:** Javier Abad, Luis Castejon, Jesus Cuartero, Roberto Palacin

**Affiliations:** 1 Department of Mechanical Engineering, University of Zaragoza, C/María de Luna s/n, 50018 Zaragoza, Spain; javabad@unizar.es (J.A.); jcs@unizar.es (J.C.); 2 School of Engineering, Newcastle University, Room 4.17, Stephenson Building, Newcastle upon Tyne NE1 7RU, UK; roberto.palacin@ncl.ac.uk

**Keywords:** bicycle handlebars, braided-carbon fiber, vibration response

## Abstract

The comfort and safety of a cyclist are directly influenced by the vibrational behavior of the handlebar. Hence, the objective of this article is to comparatively assess the vibrational characteristics of two bicycle handlebars: one made of steel and the other made of braided composite material. The transmissibility function represents the relationship between the excitation applied to both handlebars through their stems and the corresponding response in the handle area, which was experimentally obtained by applying a random vibrating signal (constant amplitude of 0.01 g^2^/Hz) using a shaker. This signal was applied in a frequency range between 100 Hz and 1200 Hz, and the response was measured at one of the two cantilevered ends of the handlebar. Different sensors, including a laser vibrometer and a control accelerometer in the shaker, were utilized. The transmissibility, natural frequencies and damping functions were obtained. Subsequently, another experimental analysis was carried out with the instrumented handlebars mounted on a bicycle, placing three accelerometers and a GPS meter and traveling through a real test circuit, with a rough surface, speed bumps and areas with shaped warning bands. Power Spectral Density (PSD) curves were obtained for the steel and carbon-fiber-composite handlebars in order to quantify the signal intensity. Finally, a fatigue analysis was carried out in order to evaluate the expected life of both handlebars under the experimentally applied load, which is considered the reference cycle. This study offers a comparative analysis of the vibration behavior exhibited by steel and carbon-fiber-composite bicycle handlebars under experimentally applied load. In conclusion, data on natural frequencies, damping functions and fatigue life expectancy for both handlebar materials were obtained. Our study provides valuable insights into the vibrational behavior and performance characteristics of steel and carbon-fiber-composite bicycle handlebars, contributing to the understanding of their comfort and safety implications for cyclists.

## 1. Introduction

The bicycle handlebar is an essential component for proper handling. Traditionally, this component is made of steel. However, on high-performance bicycles, handlebars can be made of aluminum or even a composite material of carbon fiber [1] and epoxy resin. These advanced materials offer significant advantages. Weight savings directly contribute to better dynamic performance, requiring less drive force for the same acceleration. Additionally, they exhibit improved behavior in responding to vibrations, thanks to their greater damping capacity. Lépine, J et al. [2] state that the handlebar and fork are the preponderant components of the vibration induced to the cyclist measured at their hands. In conclusion, a carbon-fiber bicycle handlebar exhibits superior resistance to fatigue loads compared to its steel counterpart. This is attributed to the carbon-fiber-composite material, which experiences much lower loss of mechanical resistance properties over a higher number of applied stress cycles than steel [3].

Various sensors can be employed to experimentally measure the response of the bicycle and the cyclist to evaluate vibrational comfort. Vanwalleghem, J. et al. [4,5] conducted tests by instrumenting the bicycle with accelerometers, custom-made contact force sensors and velocity sensors. They asserted that their sensor design approach, particularly for outdoor testing with racing bicycles, could offer a novel interpretation in evaluating cyclist comfort. This approach quantifies vibrational load not only in terms of acceleration but also in terms of force and velocity at the bicycle–cyclist contact points. Additionally, they used a strain-gauge-based handlebar force sensor that was implemented for static and dynamic load measurements. In this article, we first obtained the transmissibility function numerically, representing the relationship between excitation introduced to both handlebars through their stem and the response in the handle area. Subsequently, an experimental analysis was conducted wherein a random vibrating signal of constant amplitude (0.01 g^2^/Hz) was applied using a shaker in a frequency range between 100 Hz and 1200 Hz, and the response was measured at one of the two cantilevered ends of the handlebar. A laser vibrometer and control accelerometer in the shaker were applied. The transmissibility, natural frequencies and damping functions were obtained. Subsequently, another experimental analysis was carried out with the instrumented handlebars mounted on a bicycle, placing three accelerometers and a GPS meter and traveling through a real test circuit.

To compare the fatigue behavior of a bicycle handlebar, Jiang, CP et al. [6] employed a procedure involving a uniform design of experiments, the Kriging interpolation, the genetic algorithm and nonlinear programming methods. This procedure was applied to ABAQUS 6.22 FEM software and aligned with the ISO 4210-2:2023 [7] bicycle handlebar stem testing standard. In this paper, we compare the fatigue behavior between the original steel handlebar and the alternative carbon-fiber composite. This novel procedure involved defining the PSD (Power Spectral Density) for each handlebar and applying a Cycle Count Histogram. Fatigue damage was calculated based on accelerations rather than stresses. This novel procedure in fatigue calculation allows for a comparison of values obtained for both handlebars, enabling an inference regarding which handlebar has a longer fatigue life or less accumulated damage.

## 2. Handlebar Definition

The handlebar chosen for this study represents a commonly available model in the market, tailored for use with both MTB (Mountain Bike) and gravel bicycles. Selected for its popularity and widespread usage among riders, this handlebar is constructed from robust steel, making it well suited for the challenging demands of off-road terrains. With dimensions of 620 mm in length, a stem diameter of 25.4 mm and grips with a diameter of 22 mm and a thickness of 2.25 mm at the ends, the handlebar’s geometric characteristics play a crucial role in enhancing rider comfort and control. Noteworthy features include a back camber angle of 6° and a top bend angle of 5°. These design elements collectively contribute to improved ergonomics, providing riders with optimal handling during diverse cycling conditions. By utilizing this widely recognized and mass-marketed handlebar [8], our research aims to derive insights applicable to a broad audience of cyclists engaged in MTB and gravel biking.

**Figure 1:**
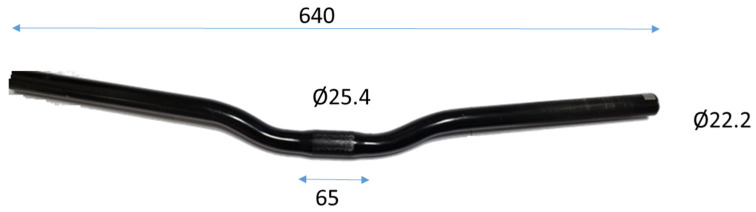
Steel handlebar (dimensions in mm).

**Figure 2:**
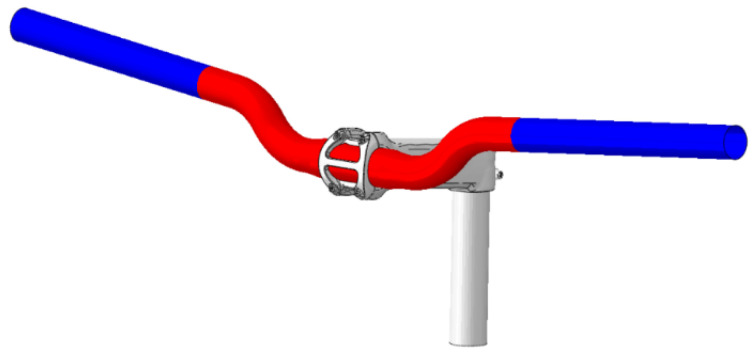
Layer zones of composite handlebar. Four layers of braiding at central part (red color) and three layers of braiding throughout rest of handlebar (blue color).

After the study of the steel handlebar as a reference, shown in Figure 1, our analysis was extended to explore additional options, with the first being the steel handlebar weighing 630 g. Subsequently, we scrutinized a cutting-edge alternative, a composite handlebar shown in Figure 2. This advanced handlebar incorporates an intricate design, featuring four layers of braiding at the central part (4.2 mm) and three layers of braiding throughout the rest of the handlebar (3.15 mm). Impressively, despite its complex construction, the braided-carbon handlebar weighs a mere 136 g.

This lightweight yet durable composition positions the braided-carbon handlebar as an intriguing alternative to traditional steel counterparts. As we delve into our analysis, we aim to assess how these structural differences impact not only the weight but also crucial factors such as strength, flexibility and overall performance. By systematically comparing these diverse handlebar materials, our research aims to offer valuable insights into the considerations that riders should weigh when selecting the most suitable handlebar for MTB and gravel biking applications.

The juxtaposition of the steel handlebar and the innovative composite handlebar allows for a comprehensive evaluation of their respective merits and drawbacks. Such insights are instrumental for cyclists seeking optimal performance and comfort in varied riding conditions.

The properties of the carbon-fiber braid (12 k) used are shown in Table 1.

## 3. Static Handlebar Calculations

Static handlebar calculations were conducted to comprehensively assess the performance and structural integrity of the handlebars under various loads. This analysis aimed to provide valuable insights into their stability and durability. Key aspects of the calculations included:

- Boundary Conditions: The base of the testing support was fixed with vertical displacement restrictions to simulate a stable foundation. Additionally, distributed loads were applied at both ends of the handlebar to replicate real-world loading conditions.
- Finite Element Model: The finite element model comprised approximately 25,000 nodes and 25,000 quadrilateral shell elements. Each section of the laminate was meticulously modeled to ensure accurate alignment with the fiber orientation and thickness.
- Nonlinear Approach: Given the potential complexity of the computations, a nonlinear approach was adopted for the finite element analysis. This allowed for the accommodation of potential large displacements and material degradation, resulting in a more realistic simulation of the handlebar’s behavior under varying loads.
- Modeling Considerations: To accurately capture the braiding orientation on the handlebar, six unique sections were utilized, each representing different combinations of diameter, thickness and fiber orientation. Modeling the stem and testing supports together was crucial due to their nonlinear behavior, ensuring that displacements were accurately represented. Conversely, the extreme sections of the handlebar were treated as entirely elastic to simplify the analysis in those areas.

These static handlebar calculations were instrumental in comparing the stiffness and strength between the steel and braided-carbon composite handlebars, providing valuable data for understanding their structural performance. Our primary strength benchmark is set at achieving a target force of 2000 N applied at each extreme of the handlebar, representing the critical threshold for the first ply failure. It is a rough estimate representing the order of magnitude in which the maximum strength of handlebars should ideally be. This value is not supported by regulation or manufacturer mandates but rather serves as a reference point based on observations of the steel handlebar’s behavior, which was able to withstand forces up to 3000 N despite losing linearity at 1800 N.

### 3.1. Strength Comparison

The strength comparison involves scrutinizing how each handlebar material withstands the applied force, with a particular focus on identifying the point of failure. This analysis will provide valuable insights into the material’s ability to endure stress and resist deformation.

The onset of first ply failure typically occurred at around 1.9–2 kN, resulting in a peak load. Despite this initial failure, the handlebars continued to bear the load until the final failure. The analyzed composite handlebars experienced catastrophic failure at the laminate joint between the central part and the rest of the bar.

### 3.2. Stiffness Evaluation

Stiffness, a key factor influencing the handlebar’s responsiveness, is examined to gauge the bending stiffness exhibited under the applied loads. This evaluation will shed light on how each handlebar material contributes to the overall rigidity and control during riding.

### 3.3. Displacement Analysis

By enforcing an absolute restriction of displacement at the stem, we aim to understand how well the handlebars maintain their form and structure under the applied loads. This aspect is integral to assessing the handlebars’ stability and their capacity to withstand external forces without compromising rider safety.

Table 2 delves into the detailed results of these static handlebar calculations, providing a comprehensive overview of the structural performance of both the steel and braided-carbon composite handlebars. This thorough examination is instrumental in guiding cyclists toward an informed choice based on the desired attributes for their MTB and gravel biking experiences.

## 4. Braided Carbon Fiber–Epoxy Resin Composite Handlebar Manufacturing

The production of the Braided Carbon Fiber–Epoxy Resin Composite Handlebar involved a meticulous process to ensure structural integrity and optimal performance.

### 4.1. Mold and Compaction Pressure

A custom mold and an internal bag for the tubes were employed. These components were instrumental in achieving compaction pressure, a critical step to ensure uniformity and strength in the final product. The mold provided the necessary shape and form for the handlebar, while the internal bag facilitated compaction by applying pressure to the composite materials.

### 4.2. Preform Preparation

**Figure 3:**
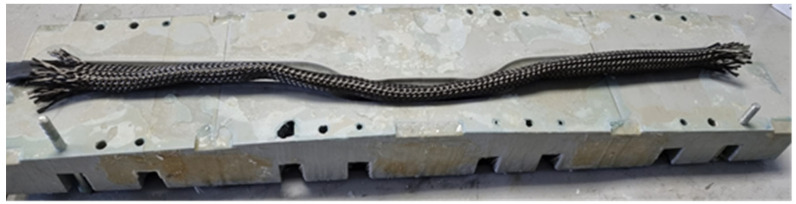
Manufacturing of braided-carbon handlebar.

To commence the manufacturing process of a handlebar, the carbon-fiber braiding had to first be cut, utilizing the mold as a reference, as shown in Figure 3. Subsequently, all layers had to be carefully unrolled to facilitate the placement of the internal bag for positive pressure compaction. These preforms, shaped according to the design specifications, served as the foundational elements for the handlebar’s structure.

### 4.3. Impregnation and Molding

Each layer was then meticulously wetted out through hand lay-up once the bag was properly positioned. Additionally, compaction was achieved using rollers to ensure uniformity and proper adherence of the layers during the manufacturing process, to enhance adhesion and overall durability. Subsequently, the impregnated preforms were carefully placed in the closed mold, ensuring proper alignment and distribution of materials. The closed mold provided a controlled environment for the molding process.

### 4.4. Internal Pressure Application

**Figure 4:**
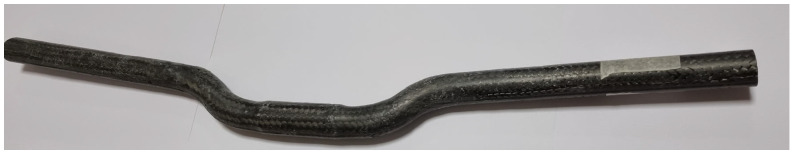
Final braided-carbon handlebar.

Upon completion of the wetting process for all layers, the mold is closed, and the bag is inflated to a pressure of up to 2 bars. Maintaining optimal pressure is crucial to achieving the desired fiber percentage and compaction, as well as ensuring the correct shaping of the mold’s curves. The pressure bag primarily consists of 80% Rubber-Isobutylene-isoprene rubber (IIR)-Butyl, supplemented with either 20% or 10% IIR. Typically, there is no necessity to utilize wax or any demolding agent, as the internal bag can be easily removed thereafter. This step ensured that the composite materials conformed precisely to the mold’s shape, eliminating voids and enhancing the structural integrity of the handlebar. The final braided-carbon handlebar can be observed in Figure 4.

This meticulous manufacturing process guarantees a high-quality Braided Carbon Fiber–Epoxy Resin Composite Handlebar with consistent performance characteristics.

The fiber volume was approximately 55–60% due to both the initial compaction with rollers and the subsequent internal pressure of 2 bars. Several tests were conducted, with a maximum pressure of 6 bars, but to achieve optimal compaction and curing, a final decision was made to use a pressure of 2 bars and a curing time of 24 h at 50 °C.

## 5. Natural Frequency Calculation

In pursuit of understanding the dynamic behavior of the two handlebars under consideration, a comprehensive analysis of their natural frequencies and vibration modes was conducted. This process involved the creation of a numerical model implemented in ABAQUS, incorporating specific geometrical and material properties.

### 5.1. Numerical Model Configuration

The numerical model was configured to reflect the distinct material properties of each handlebar. For the steel handlebar, a linear isotropic material model was employed, while the handlebar manufactured using carbon fiber–epoxy utilized a linear orthotropic material model to accurately represent the anisotropic nature of the composite material.

### 5.2. Boundary Conditions

Consistent with the conditions applied in the static calculations, the same boundary conditions were adopted for the natural frequency analysis. The central part of the handlebars was set over a width of 20 mm, ensuring alignment with real-world scenarios and facilitating a meaningful comparison of results.

### 5.3. Modal Analysis and Lanzcos Algorithm

**Figure 5:**
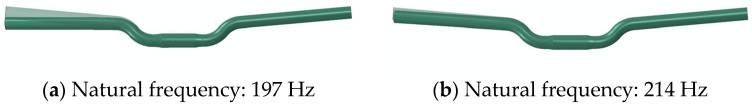
Natural frequency and vibration natural mode for the steel handlebar (**a**) and the braided-carbon handlebar (**b**).

Natural frequencies were determined through modal analysis, employing the Lanzcos algorithm [9]. This algorithm is well suited for extracting modal information, providing an accurate depiction of vibration modes and associated natural frequencies. Figure 5 showcases the modal shape of the first mode, offering a visual representation of the handlebar’s response to dynamic forces.

### 5.4. Adjustment of Test Parameters

The information derived from the natural frequency calculation serves as a crucial foundation for adjusting the parameters of subsequent tests. By understanding the handlebars’ dynamic characteristics, the testing approach could be refined to ensure a comprehensive evaluation of their performance in real-world cycling conditions.

The modal shapes and associated natural frequencies, as depicted in Figure 5, will be further analyzed to draw meaningful insights into the dynamic behavior of each handlebar. This analysis contributes valuable data for cyclists seeking handlebars that not only excel in static scenarios but also demonstrate optimal vibrational characteristics during various riding conditions.

## 6. Dynamic Test of the Two Handlebars

To analyze the dynamic behavior of the steel and Braided Carbon Fiber–Epoxy Resin Composite Handlebars, a comprehensive dynamic test was conducted using experimental methods. The transmissibility function, quantifying the relationship between the excitation introduced to the handlebar and the resulting response in the grip area, was determined.

### 6.1. Experimental Setup

Each handlebar, whether made of steel or the Braided Carbon Fiber–Epoxy Resin Composite, was equipped with a shaker rigidly fixed to its central part using a flange. This shaker facilitated the application of a random vibrating signal with a constant amplitude of 0.01 g^2^/Hz in a frequency range spanning from 100 Hz to 1200 Hz.

The term “0.01 g^2^/Hz constant amplitude” refers to the magnitude of the vibrating signal applied during our experimental testing procedures. Specifically, it represents the amplitude of acceleration measured in gravitational units (g) squared per Hertz (Hz) of frequency bandwidth. This parameter signifies the intensity of the vibration stimulus exerted on the handlebars across varying frequencies.

To determine the frequency transfer functions in the handlebars, a random vibration is applied to the base defined by a PSD (Power Spectral Density) curve of constant amplitude within the frequency range between 100 Hz and 1200 Hz. In this context, the entire frequency range is excited with the same amplitude. The units of the PSD are square acceleration divided by Hertz.

### 6.2. Response Measurement

The response to the applied excitation was measured at one of the two cantilevered ends of the handlebar using a laser vibrometer. This high-precision instrument allowed for accurate detection and quantification of the vibrational characteristics at the specified locations.

### 6.3. Transmissibility Function

By analyzing the transmissibility function between the excitation and response, critical insights into the dynamic characteristics of each handlebar were obtained. This function facilitated the determination of resonant frequencies and damping for both the steel and Braided Carbon Fiber–Epoxy Resin Composite Handlebars.

### 6.4. Damping Measurement

The damping of each handlebar was quantified using the half-bandwidth method [10]. This method, applied to the transmissibility functions obtained for each handlebar, provided a clear understanding of the energy dissipation characteristics during dynamic loading.

### 6.5. Sensor Configuration

To ensure precise control and measurement, a closed-loop system was implemented, employing a control accelerometer in the shaker. Automatic regulation and correction mechanisms were applied to maintain the response equal to the setpoint in terms of acceleration.

**Figure 6:**
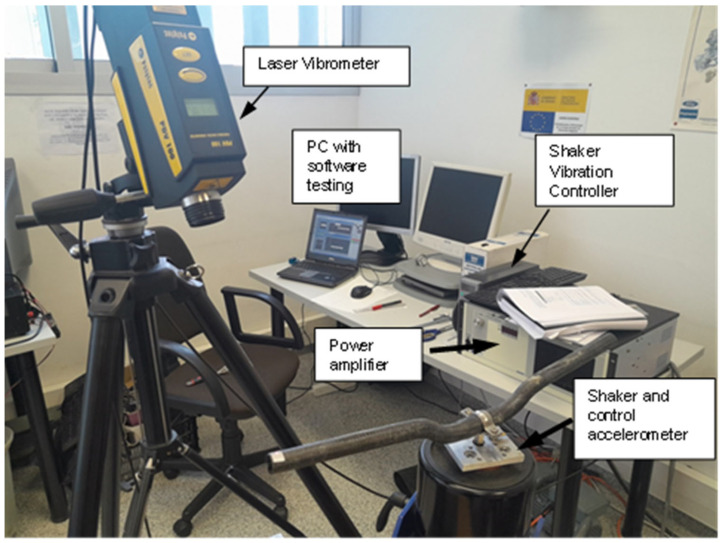
Testing arrangement for natural frequencies and damping determination.

This meticulous dynamic testing approach, combining controlled excitation, precise response measurement and advanced sensor configurations, enables a thorough assessment of the vibrational characteristics of both handlebars. This testing arrangement can be seen in Figure 6.

### 6.6. Experimental Modal Analysis (EMA)

**Figure 7:**
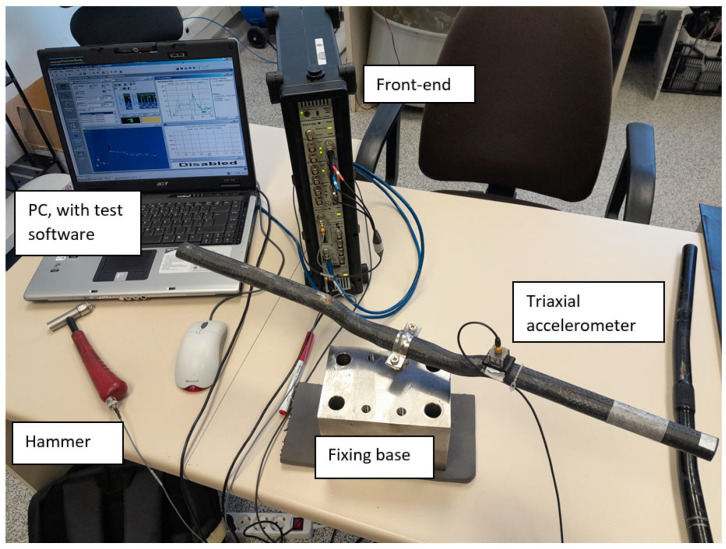
Equipment used for the Experimental Modal Analysis (EMA).

The first mode and natural frequency of the two configurations analyzed were experimentally determined. For this purpose, an Experimental Modal Analysis (EMA) was conducted using an impact hammer to excite the structure and a low-mass triaxial accelerometer to measure the response. The handlebar was rigidly fixed in the central part to replicate the boundary conditions of the numerical modal analysis. Figure 7 illustrates the equipment used and the test configuration.

### 6.7. Transfer Functions

**Figure 8:**
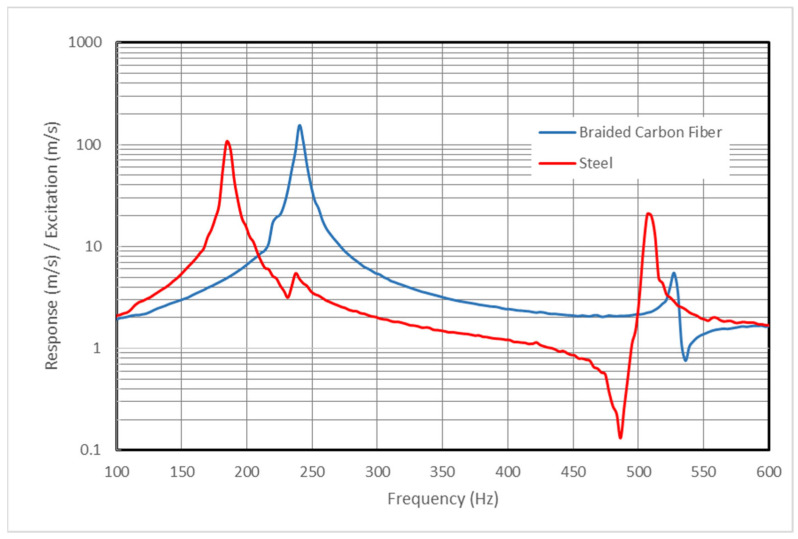
Transfer functions for steel and braided-carbon-fiber handlebars.

The transfer functions obtained for the two handlebars under testing, one constructed from steel and the other from Braided Carbon Fiber–Epoxy Resin Composite, are visualized in Figure 8.

In the analysis of these transfer functions, it is evident that the braided-carbon-fiber handlebar exhibits greater specific stiffness (stiffness divided by weight) compared to its steel counterpart. Notably, the carbon-fiber handlebar demonstrates lower damping, emphasizing its distinct dynamic characteristics.

Table 3 summarizes key parameters for the first mode of vibration under the test conditions.

The data illustrate the marked differences between the steel and braided-carbon-fiber handlebars. The carbon-fiber handlebar, with its lower mass and higher natural frequency, exemplifies enhanced stiffness. Meanwhile, the steel handlebar demonstrates comparatively higher damping.

## 7. Vibrational Comfort Evaluation

To assess the vibratory comfort of each handlebar configuration under analysis, the methodology outlined in the ISO 5349-1 (2001) standard, ref. [11] titled “Mechanical Vibration-Measurement and Evaluation of Human Exposure to Hand-Transmitted Vibration”, was employed. This standard provides a framework for evaluating the impact of vibrations on human comfort and well-being.

**Figure 9:**
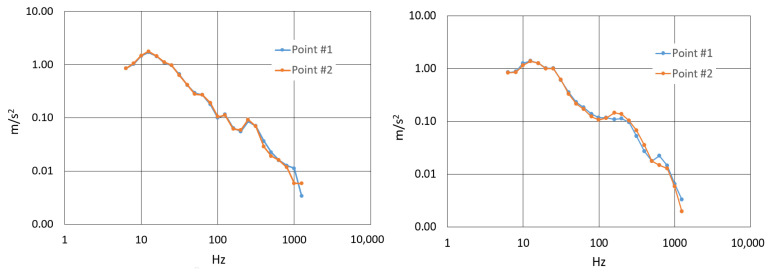
One-third-octave-frequency-weighted R.M.S. acceleration spectrum. (**Left**): Steel handlebar. (**Right**): Carbon-fiber handlebar.

For this evaluation, the total vibration value of frequency-weighted Root Mean Square (R.M.S.) acceleration was determined for both the steel and carbon-fiber handlebars. Acceleration spectra were recorded at points #1 and #2, corresponding to the ends of the handlebars where the cyclist’s hands rest. Vibration signals were captured along the three Cartesian axes: X, Y and Z. Refer to Figure 9 for visual representations of the acceleration spectra.

To provide a quantitative assessment of comfort, the R.M.S. acceleration values were calculated for the frequency-weighted hand-transmitted vibration. The results are summarized in Table 4 below.

Lower values of R.M.S. (Root Mean Square) acceleration indicate better comfort. A lower R.M.S. acceleration signifies the reduced intensity of overall vibration experienced by the cyclist’s hands, contributing to a more comfortable and less fatiguing riding experience. Therefore, the lower R.M.S. acceleration values for the carbon-fiber handlebar compared to the steel handlebar suggest that the carbon-fiber handlebar may offer better vibrational comfort to the cyclist.

## 8. Track Test: Assessing On-Bike Performance

**Figure 10:**
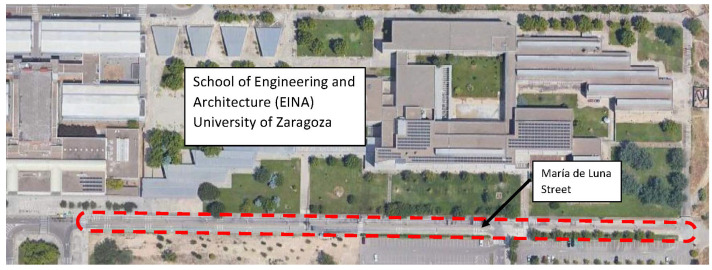
Layout and location of test track. The test circuit is marked in red color.

To comprehensively evaluate the behavior of the handlebars when integrated into the cycling experience, a field test was executed on a circuit adjacent to the EINA at the University of Zaragoza. The test circuit, as depicted in Figure 10, comprises two straight sections, each with a length of 375 m, as well as two curved sections, and encompasses a variety of terrain challenges.

**Figure 11:**
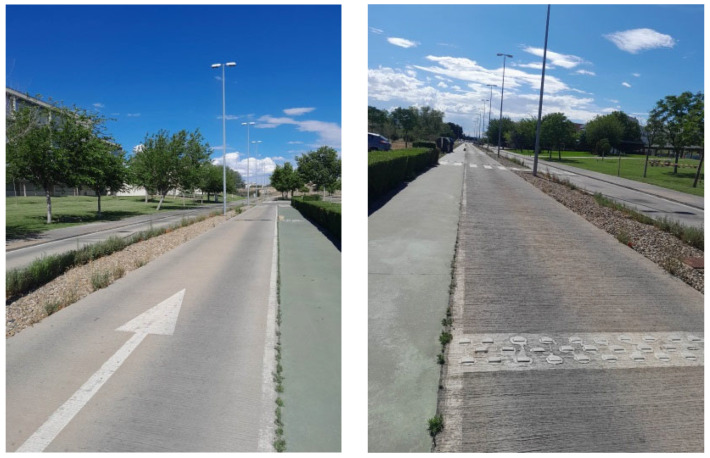

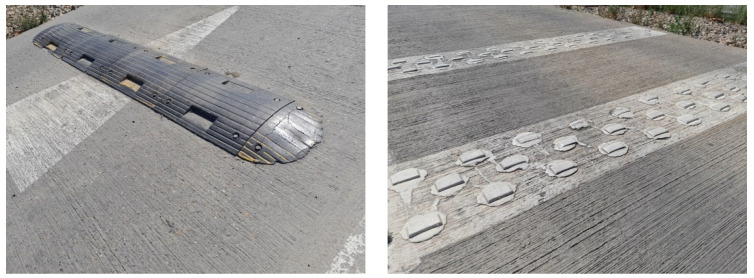
Images of track and details of obstacles.

The circuit not only serves as an open space for dynamic assessments but also incorporates features to simulate real-world cycling scenarios. Notably, the track includes a rough surface and strategically positioned obstacles, such as 7 speed bumps and 15 zones with warning strips, as illustrated in Figure 11.

The track test involved a total of four measurements, with two conducted using the steel handlebar and two using the carbon-fiber handlebar. This approach ensures a comprehensive understanding of how each handlebar variant performs in the face of varied challenges encountered during cycling.

To rigorously test and assess the performance of each handlebar once integrated into the bicycle, a comprehensive set of sensors was strategically applied. The instrumentation aimed to capture key dynamic parameters and provide valuable insights into the behavior of the bicycle under real-world riding conditions.

### 8.1. Accelerometer Configuration

**Figure 12:**
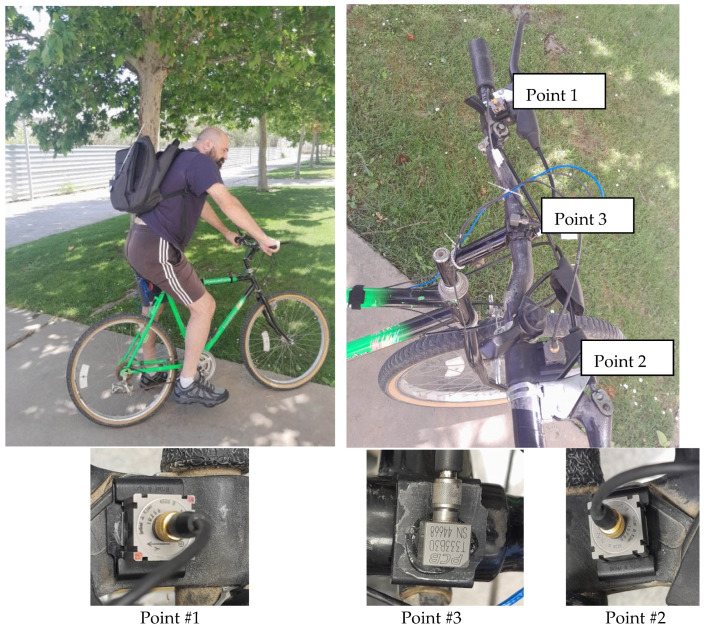
Bicycle rider equipped with backpack to carry LMS SCADAS RECORDER equipment and detailed view of three accelerometers strategically positioned on bicycle handlebar.

Three accelerometers were strategically positioned to capture dynamic responses. Refer to Figure 12.

Triaxial Brüel Accelerometers:

- Positioned at the ends of the handlebars in the lever area (Point #1 and Point #2).
- Designed to precisely measure accelerations along three axes, offering detailed insights into handlebar dynamics.

PCB Accelerometer:

- Mounted on the handlebar stem (Point #3).
- Provides critical data regarding the vibrational characteristics experienced by the seat during cycling.

### 8.2. Speed Measurement

- A GPS sensor was employed to measure the speed of the bicycle.
- This sensor allowed for accurate recording of the bicycle’s speed, contributing to a comprehensive understanding of its dynamic behavior.

### 8.3. Recording Equipment

- The LMS SCADAS RECORDER equipment was utilized to record and store the measurements. Refer to Figure 12.
- The software Simcenter Testlab 2206 version 2206.0001, from SIEMENS, was applied for the analysis and processing of registered measurements.
- Known for its reliability and precision, this equipment ensured the accurate capture of dynamic data during the bicycle testing.

The findings from the track test contribute essential insights into the practical implications of the handlebars’ dynamic characteristics. The instrumentation deployed for bicycle testing reflects a meticulous approach to gathering comprehensive data on the behavior of each handlebar in real-world cycling scenarios. The combination of accelerometers at key points and a GPS sensor provides a holistic perspective, allowing for a detailed analysis of the bicycle’s dynamics and handlebar performance.

**Figure 13:**
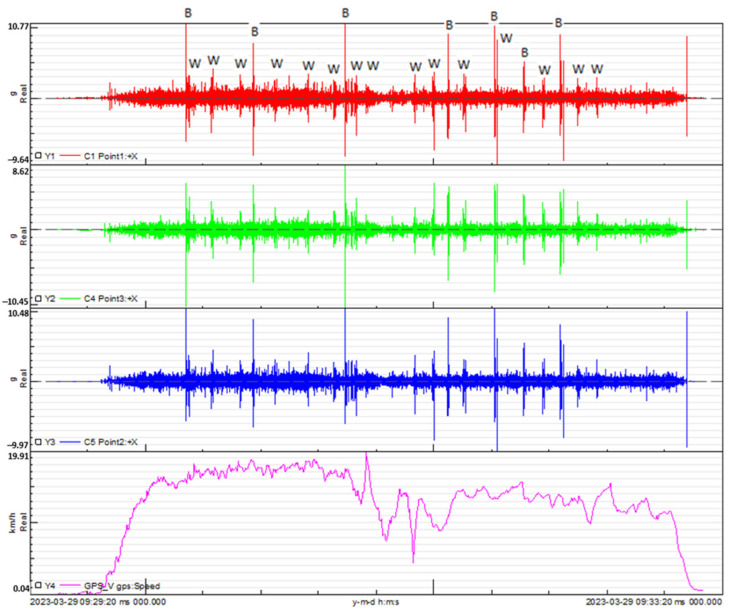
Vertical acceleration measured at points 1, 2 and 3, corresponding to the locations of the applied accelerometers and the speed versus time for the steel handlebars. (B) Location of the 7 speed bumps and (W) location of the 15 warning strips.

The vibration signals were meticulously recorded during the track test, employing a high sampling frequency of 10.240 Hz. Concurrently, the speed signal was captured using a GPS device, sampled at a frequency of 4 Hz. In Figure 13, the outcomes of the track test for the steel handlebar are presented. The graphs portray the vertical acceleration measured at points 1, 2 and 3, corresponding to the locations of the applied accelerometers. Additionally, the relationship between speed and time is illustrated, providing a comprehensive visualization of the handlebar’s performance under real-world cycling conditions.

**Figure 14:**
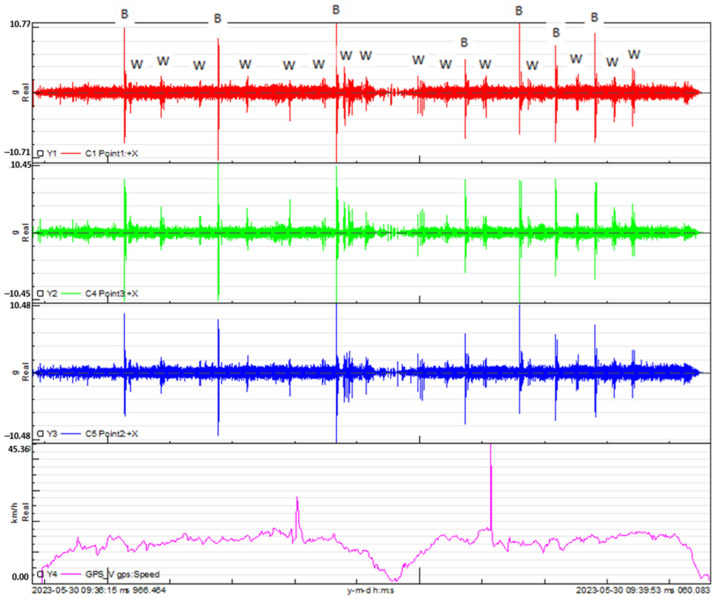
Vertical acceleration measured at points 1, 2 and 3, corresponding to the locations of the applied accelerometers and the speed versus time for the braided-carbon-fiber-composite handlebar. (B) Location of the 7 speed bumps and (W) location of the 15 warning strips.

Figure 14 showcases the outcomes from the track test for the braided-carbon-fiber-composite handlebar. The graphs display the vertical acceleration measured at points 1, 2 and 3, corresponding to the locations of the applied accelerometers. Additionally, the relationship between speed and time is depicted, providing a comprehensive visualization of the braided-carbon-fiber handlebar’s performance during the track test.

In Figure 13 and Figure 14, the observed trend reveals that the highest acceleration peaks align with the bicycle passing over large bumps, whereas the lowest acceleration peaks correspond to smaller bumps. Leveraging the tests conducted on the circuit and the recorded measurements of acceleration at key points on the bicycle, coupled with speed versus time data, a Power Spectral Density (PSD) [12] analysis was performed for each handlebar. This analysis entails measuring acceleration squared per Hertz versus frequency, where the square root of the area under the curve provides the Root Mean Square (RMS) value. This metric serves to quantify the signal intensity, offering valuable insights into the dynamic response of each handlebar during real-world cycling conditions.

**Figure 15:**
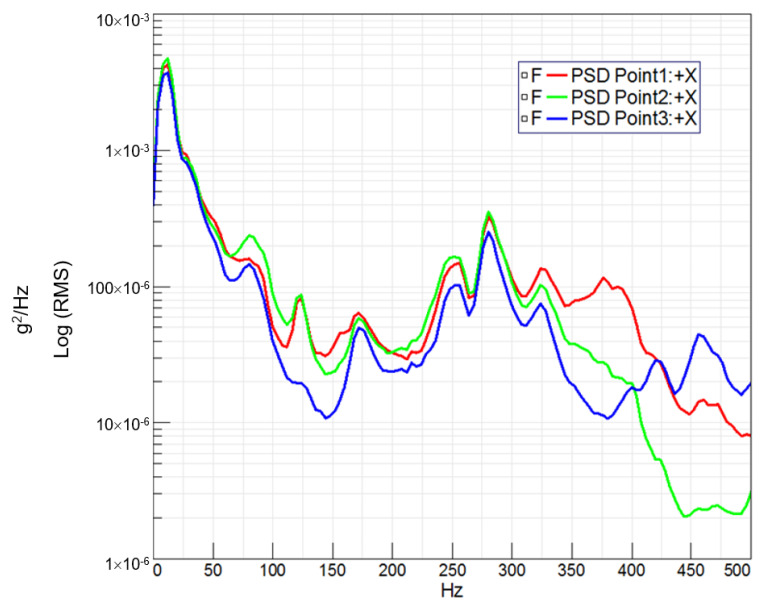
PSD (Power Spectral Density) (g^2^/Hz) vs. frequency (Hz) obtained for the steel handlebar at three acceleration measurement points on the handlebar.

**Figure 16:**
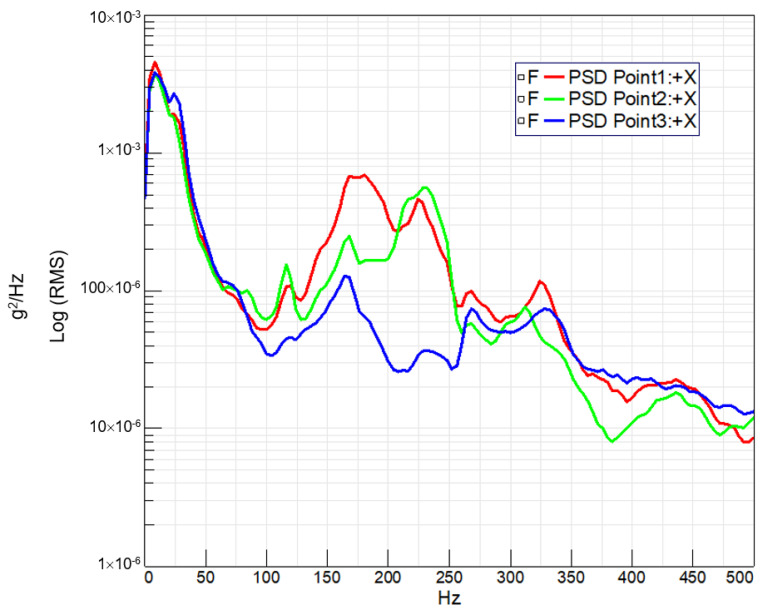
PSD (Power Spectral Density) (g^2^/Hz) vs. frequency (Hz) obtained for the braided-carbon-fiber-composite handlebar at three acceleration measurement points on the handlebar.

In both Figure 15 and Figure 16, which depict PSD analyses, the initial peak below 20 Hz is attributed to the bicycle wheel’s suspension. However, subsequent peaks in these curves reflect the vibrational response of the handlebar structure.

Furthermore, it is noteworthy that a second resonance peak emerges in the steel handlebar above 75 Hz, absent in the carbon handlebar. Conversely, the composite handlebar exhibits resonance peaks of greater amplitude between 150 Hz and 250 Hz, aligning with its higher natural frequency and lower damping characteristics.

## 9. Fatigue Analysis

In order to analyze the fatigue life of the steel handlebar and determine pseudo-damage, it is crucial to identify the number of complete vibration cycles occurring between maximum and minimum acceleration values. This information is extracted from the temporary acceleration signals measured at various points along the handlebar. To achieve this, a cycle counting technique [13] known as rainflow analysis is employed. This method enables the determination of how many times a specific vibration cycle, characterized by its maximum and minimum values, repeats within the temporary signal.

**Figure 17:**
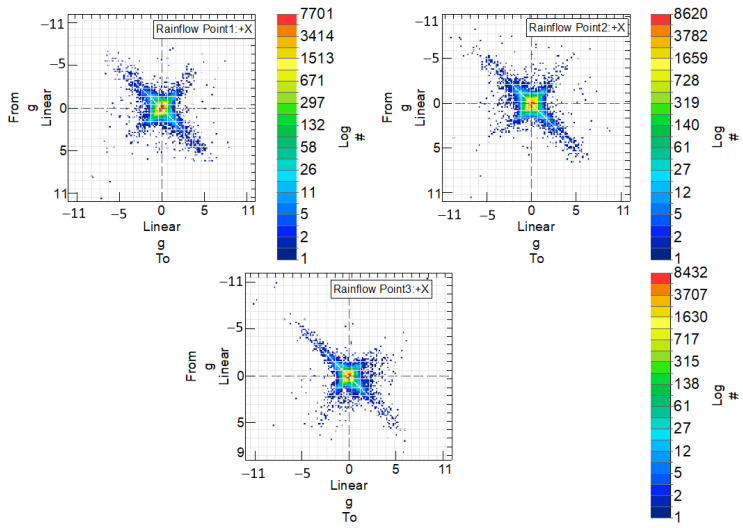
Points corresponding to cycles measured between extreme acceleration values, for the steel handlebar, at the three measurement points at which accelerometers were applied.

Figure 17 illustrates the number of cycles measured between extreme acceleration values for the steel handlebar at the three designated measurement points where accelerometers were applied. The legend “from” and “to” in the graph represent the initial and final values, respectively, defining the vibration cycle. The color scale indicates the frequency of occurrence of these vibration cycles, with each color representing a different number of cycles. To enhance clarity, the values on the color scale are represented on a logarithmic scale denoted by the legend “log”.

Points located along the bisector at +45° signify positions where no vibration occurs. These points represent acceleration values that remain constant, transitioning from a certain value to the same value. Conversely, points outside this bisector indicate vibration, as they oscillate between different acceleration values.

**Figure 18:**
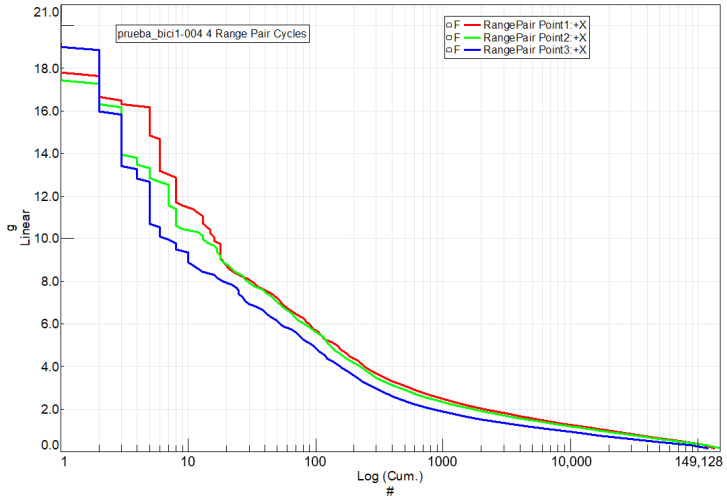
A histogram counting the vibration cycles for the steel handlebar at each of the three acceleration measurement points on the handlebar. Y-axis: Range of acceleration variation (g) vs. X-axis: Sum of cycles.

Figure 18 presents the histogram that tallies the cycles represented in Figure 17 for the steel handlebar at each of the three acceleration measurement points. A total of 149,128 cycles were counted. The histogram illustrates the distribution of cycles across various ranges of acceleration variation.

In the culmination of this analysis, a critical measure of pseudo-damage arising from the maneuvers executed on the circuit is introduced. Notably, this pseudo-damage is computed based on accelerations rather than tensions, providing a unique perspective on the structural response of the handlebars. Module Testlab Neo Durability from Simcenter Testlab 2206 version 2206.0001 software was applied.

The expression for pseudo-damage is derived from the accelerations observed during the maneuvers, and it is paramount in quantifying the potential structural implications on the handlebars. It is important to note that, in some contexts, an equivalence can be drawn between applied acceleration and induced tension. This equivalence serves as a valuable reference, shedding light on the structural implications of the dynamic forces encountered during the circuit maneuvers.

The expression for pseudo-damage derived from accelerations can be based on cumulative fatigue damage models. One commonly used model is Miner’s Rule [14,15,16], which assumes that damage accumulates linearly with the number of cycles and that the structure fails when the cumulative damage reaches a critical value.

Let us denote *D_i_* as the pseudo-damage at the *i*-th cycle and *N_i_* as the number of cycles at that specific acceleration level. The pseudo-damage *D_i_* at each cycle is calculated by dividing the actual number of cycles (*n_i_*) by the corresponding endurance limit ($N_{i}^{e}$) at that acceleration level.

The expression for pseudo-damage (*Di*) can be given as

(1) $$D_{i}=\frac{N_{i}}{N_{i}^{e}}$$

Here, *N_ie_* represents the endurance limit at the *i*-th acceleration level. The total pseudo-damage (*D_total_*) accumulated over all cycles is then the sum of the pseudo-damage at each acceleration level:

(2) $$D_{total}=\sum_{i=1}^{k}D_{i}$$

where k is the total number of different acceleration levels observed during the maneuvers.

The S-N curve used for S355 steel was the one available in the Testlab Neo Durability database, considering a tensile strength of 450 MPa and a fatigue stress limit equal to 205 MPa [17].

**Figure 19:**
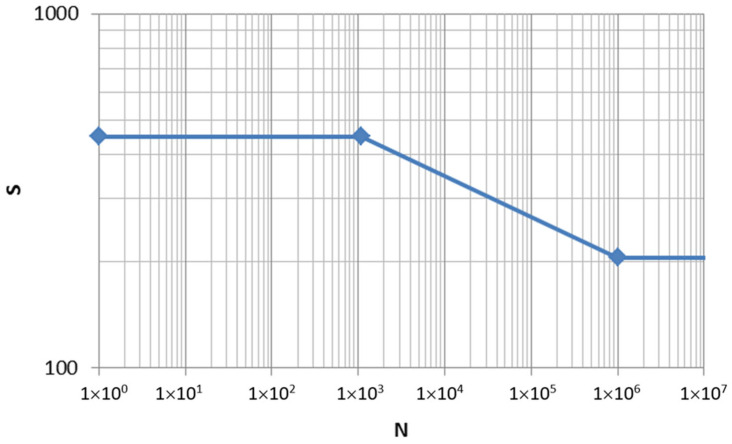
S-N curve used for S355 steel obtained from Testlab Neo Durability database.

By means of the application of the S-N curve shown in Figure 19, the pseudo-damage shown in Table 5 was obtained for the steel handlebar. In this figure, stress amplitude corresponds to a stress ratio (R = −1), and N is cycles to failure.

**Figure 20:**
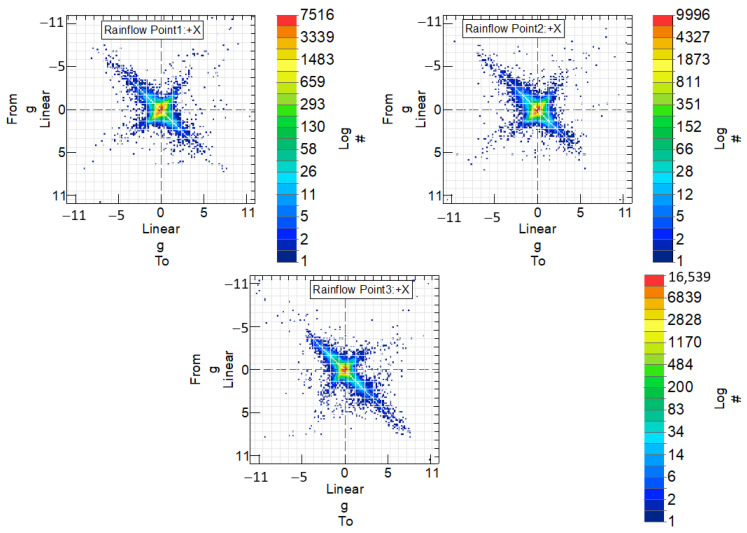
Points corresponding to cycles measured between extreme acceleration values, for the braided-carbon-fiber-composite handlebar, in the three measurement points in which accelerometers were applied.

**Figure 21:**
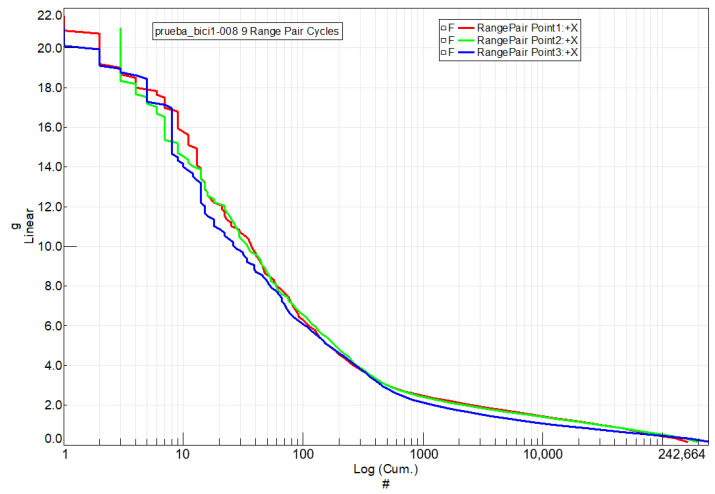
A histogram counting the vibration cycles for the braided-carbon-composite handlebar at each of the three acceleration measurement points on the handlebar. Y-axis: Range of acceleration variation (g) vs. X-axis: Sum of cycles.

The same comprehensive analysis was applied to the handlebar made of the braided-carbon-fiber composite. In Figure 20, the cycles between extreme speed values are presented for this handlebar at the three measurement points where accelerometers were applied. Figure 21 complements this by showcasing the histogram, illustrating the distribution of vibration cycles at each of the three acceleration measurement points on the handlebar.

**Figure 22:**
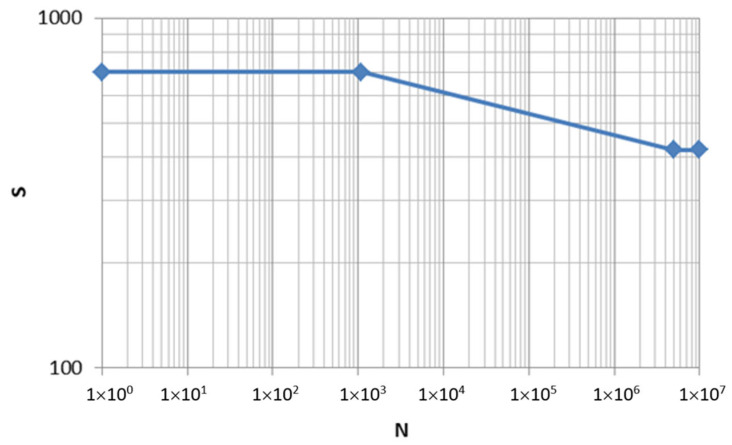
S-N curve used for braided carbon and epoxy resin composite [18].

The S-N curve used for the braided carbon and epoxy resin composite, shown in Figure 22, was obtained from the research carried out by Carvelli, V. et al. [18], taking into account that tensile strength in the longitudinal direction (X) equals 700 MPa and fatigue strength can be calculated as a 60% of X (420 MPa).

For a holistic understanding, Table 6 encapsulates the measured pseudo-damage at each of the measurement points in the handlebar, providing a quantitative assessment of the handlebar’s structural response to the dynamic conditions encountered during cycling maneuvers.

Pseudo-damage values for steel and composite handlebars summarized in Table 7 represent a pseudo-damage assessment derived from the accelerations measured during the testing process, rather than direct stresses. Pseudo-damage serves as a qualitative indicator used for comparative analysis between different vibratory situations, enabling an estimation of which scenario is more detrimental in terms of fatigue. In this case, we are comparing two different vibratory situations applied to two different handlebars.

While the numerical value itself may appear insignificant on its own, its significance lies in the comparison between different vibratory conditions. By comparing the pseudo-damage values obtained for the two analyzed vibratory situations and handlebars, we can assess which scenario is more restrictive in terms of potential fatigue. This comparison allows us to make informed decisions about the relative durability and performance of the handlebars under different vibrational loads.

In conclusion, it can be stated that the Pseudo-damage in the steel handlebar is of various orders of magnitude greater than in the braided-carbon-fiber handlebar.

## 10. Discussion

### 10.1. Material Comparison

This study compared the fatigue behavior of a traditional steel handlebar with an alternative braided-composite handlebar.

The carbon-fiber handlebar demonstrated superior resistance to fatigue loads compared to its steel counterpart.

### 10.2. Static Handlebar Calculations

Static calculations were conducted to evaluate stiffness, strength and displacement under various loads for both steel and braided-carbon-fiber handlebars.

The braided-carbon-fiber handlebar exhibited higher strength and enough stiffness compared to the steel handlebar.

### 10.3. Manufacturing Process

The production process for the braided-carbon-fiber handlebar involved meticulous steps, including mold and compaction pressure, preform preparation, impregnation, molding and internal pressure application.

### 10.4. Natural Frequency Calculation

Numerical models were used to analyze the natural frequencies and vibration modes of both handlebars.

The braided-carbon-fiber handlebar showed higher natural frequencies, indicating enhanced specific stiffness vs. weight.

### 10.5. Dynamic Tests

Dynamic tests were conducted to assess the vibrational characteristics of the handlebars.

The braided-carbon-fiber handlebar exhibited greater stiffness and lower damping than the steel handlebar.

### 10.6. Vibrational comfort

In conclusion, the vibrational comfort evaluation conducted using the methodology outlined in the ISO 5349-1 (2001) standard [11] indicates that the carbon-fiber handlebar may offer better vibrational comfort to cyclists compared to the steel handlebar.

This conclusion is supported by the lower R.M.S. (Root Mean Square) acceleration values recorded for the carbon-fiber handlebar at both measurement points #1 and #2, indicating a reduced intensity of overall vibration experienced by the cyclist’s hands. Lower R.M.S. acceleration values correspond to a more comfortable and less fatiguing riding experience, highlighting the potential benefits of using carbon-fiber handlebars in terms of vibrational comfort.

### 10.7. Track Test

Field tests were performed on a track to evaluate the on-bike performance of both handlebars.

Accelerometers and a GPS sensor were strategically placed to measure dynamic responses and speed.

Power Spectral Density (PSD) analysis revealed distinct vibrational responses for each handlebar.

### 10.8. Fatigue Analysis

A comparative analysis of the fatigue life of both handlebars was conducted using Rainflow Counting Pseudo-damage.

A critical measure of pseudo-damage was introduced, computed based on accelerations rather than tensions. This provided a unique perspective on the structural response of the handlebars. The pseudo-damage expression was derived from accelerations using cumulative fatigue damage models, such as Miner’s Rule. This model assumes damage accumulates linearly with the number of cycles until a critical value is reached. Pseudo-damage was calculated for both handlebars at different acceleration measurement points.

The conclusion highlighted that the pseudo-damage in the steel handlebar was of various orders of magnitude greater than in the braided-carbon-fiber handlebar.

In summary, the analysis provided a comprehensive understanding of the fatigue life of the handlebars, with a focus on the structural implications of dynamic forces encountered during cycling maneuvers. The results favored the braided-carbon-fiber handlebar, suggesting its superior performance in terms of fatigue resistance compared to the steel handlebar.

## 11. Conclusions

In conclusion, this research suggests that the braided-carbon-fiber handlebar outperforms the steel handlebar in terms of fatigue resistance, stiffness and overall dynamic performance, making it a promising alternative for high-performance bicycles.

## Figures and Tables

**Table 1:** Mechanical properties of carbon-fiber braid (12 k) utilized.

Property	Unit	Value
E11	MPa	60,000
E22	MPa	18,000
G12	MPa	12,500
µ		0.18
Density	kg/m^3^	1550
X	MPa	700
X’	MPa	550
Y	MPa	100
Y’	MPa	80
S12	MPa	120
Braiding yarns		12 k
Braiding angle	°	±25

**Table 2:** Maximum load and the corresponding displacement at the handlebar extreme obtained for both handlebars when failure took place.

Handlebar Material	Maximum Load (N)	Maximum Displacement (mm)	Bending Stiffness (N/mm)
Steel	1800	8	225
Braided carbon fiber–epoxy	1900	24	79.16

**Table 3:** Mass, first natural frequency and damping for first mode.

Handlebar Material	Mass (gr)	f_n_ (Hz)	Damping ζ (%)
Steel	636	184.7	1.79
Braided carbon fiber–epoxy	136	240.3	0.88

**Table 4:** R.M.S. acceleration value of frequency-weighted hand-transmitted vibration.

Measurement Point	Steel Handlebar a_hw_ (m/s^2^)	Carbon-Fiber Handlebar a_hw_ (m/s^2^)
Point #1	3.42	2.98
Point #2	3.49	3.07

**Table 5:** Pseudo-damage produced by the performed maneuver in the steel handlebar.

Acceleration Measurement Point	Rainflow Counting Pseudo-Damage
1	110.95 × 10^−39^
2	82.98 × 10^−39^
3	66.09 × 10^−39^

**Table 6:** Pseudo-damage produced by the performed maneuver in the braided-carbon-fiber-composite handlebar.

Acceleration Measurement Point	Rainflow Counting Pseudo-Damage
1	48.57 × 10^−117^
2	52.92 × 10^−117^
3	31.77 × 10^−117^

**Table 7:** Pseudo-damage produced by the performed maneuver in the steel and braided-carbon-fiber-composite handlebars.

Acceleration Measurement Point	Rainflow Counting Pseudo-Damage in Steel Handlebar	Rainflow Counting Pseudo-Damage in Composite Handlebar
1	110.95 × 10^−39^	48.57 × 10^−117^
2	82.98 × 10^−39^	52.92 × 10^−117^
3	66.09 × 10^−39^	31.77 × 10^−117^

## Data Availability

Data are contained within the article.
